# Survival Benefit of Combined Chemoimmunotherapy and Radiation Therapy in Patients with Recurrent or Metastatic Esophageal Cancer

**DOI:** 10.1016/j.adro.2025.101890

**Published:** 2025-08-22

**Authors:** Xueru Wang, Danyu Guo, Xiaoyang Li, Yuan He, Jieyong Tian, Dong Qian, Jingjing Cheng

**Affiliations:** a Department of Radiation Oncology, the First Affiliated Hospital of USTC, Division of Life Sciences and Medicine, University of Science and Technology of China, Hefei, Anhui, China; b Department of Thoracic Surgery, the First Affiliated Hospital of USTC, Division of Life Sciences and Medicine, University of Science and Technology of China, Hefei, Anhui, China; c Department of Radiation Oncology, Anhui Provincial Cancer Hospital, Hefei, Anhui, China

## Abstract

**Purpose:**

Chemotherapy combined with immune checkpoint inhibitors (ICIs) has become the standard first-line treatment for recurrent or metastatic esophageal cancer, but its efficacy remains suboptimal. This study aimed to evaluate whether the addition of radiation therapy (RT) to ICIs can improve patients’ survival.

**Methods and Materials:**

This retrospective cohort study analyzed clinical data from 178 patients with recurrent or metastatic esophageal cancer who were treated at the First Affiliated Hospital of USTC between December 2019 and October 2023. Based on their actual treatment regimens, patients were stratified into 2 groups: the chemoimmunotherapy-alone group (ICIs group) and the chemoimmunotherapy combined with RT group (ICIs + RT group). To minimize selection bias, propensity score matching was used to balance baseline characteristics between the groups before comparative analysis. The primary endpoint was overall survival, and the secondary endpoints were progression-free survival and safety.

**Results:**

After propensity score matching, 128 patients were selected for the final analysis, with 64 patients in the ICIs + RT group and 64 patients in the ICIs group. The median follow-up time was 11.26 months (95% CI, 7.02-15.32). The median overall survival was 23.71 months in the ICIs + RT group and 13.00 months in the ICIs group (hazard ratio, 0.53; 95% CI, 0.31-0.88; *P* = .013). The median progression-free survival was 10.43 months in the ICIs + RT group and 7.27 months in the ICIs group (hazard ratio, 0.61; 95% CI, 0.39-0.94; *P* = .024). Combination of chemoimmunotherapy and RT was safe and tolerable. No treatment-related deaths occurred in either group.

**Conclusions:**

Adding RT can significantly improve survival in patients with recurrent or metastatic esophageal cancer who are treated with chemoimmunotherapy, but further prospective trials are needed for validation.

## Introduction

Esophageal cancer is the seventh most commonly diagnosed cancer and the sixth leading cause of cancer-related death globally.^1^ Histologic subtypes of esophageal cancer vary by region, with esophageal squamous cell carcinoma (ESCC) being the predominant subtype in East Asia.^2^^,^^3^ More than half of all cases of esophageal cancer are diagnosed at an advanced stage, with locally advanced or metastatic lesions present. The current standard first-line treatment for recurrent or metastatic ESCC is a combined regimen that incorporates immune checkpoint inhibitors (ICIs) and chemotherapy. This treatment has been shown to confer superior outcomes in terms of overall survival (OS) and progression-free survival (PFS) compared with chemotherapy alone. However, the efficacy of this treatment (median PFS ∼6 months, median OS ∼15 months) still falls short of expectations.^4^, ^5^, ^6^, ^7^

A multitude of retrospective and prospective studies from the era of chemotherapy have demonstrated that radiation therapy (RT) can effectively relieve the symptoms of dysphagia in patients with advanced esophageal cancer, thereby improving the quality of life and nutritional status of these patients. Furthermore, palliative RT targeting the esophageal lesion has been demonstrated to significantly prolong OS in patients whose disease is well controlled after first-line chemotherapy.^8^^,^^9^ The treatment of advanced esophageal cancer has entered the era of immunotherapy, with ICIs being widely used in both first- and second-line treatments.^4^^,^^6^ Nevertheless, it remains uncertain whether the combination of RT and immunotherapy improves patient survival and whether it is safe.

Theoretically, RT and immunotherapy have synergistic effects. The Keynote Evaluating Your New Oncology Therapy Experiment (KEYNOTE)-001 study, which investigated the efficacy of pembrolizumab (an anti-programmed cell death protein 1 (anti-PD-1) therapy) in later-line treatments for various solid tumors, demonstrated that patients who had previously received RT exhibited superior outcomes with ICIs.^10^ In addition, the concurrent use of RT and ICIs is becoming increasingly prevalent in both clinical research and practice. In locally advanced esophageal cancer, neoadjuvant chemoradiotherapy combined with ICIs, as well as definitive chemoradiotherapy combined with ICIs, have demonstrated encouraging outcomes in several phase 2 studies.^11^^,^^12^ Phase 3 randomized controlled trials are also underway and are expected to change current clinical practice in the future. However, there has been limited exploration of ICIs combined with RT for recurrent/metastatic esophageal cancer, and past experiences from the chemotherapy era are insufficient to guide current clinical practice.

The aim of this study was to evaluate whether RT can provide survival benefits in patients with recurrent or metastatic esophageal cancer receiving ICIs treatment and to analyze the safety of the combined treatment.

## Methods and Materials

### Study design and participants

**Figure 1:**
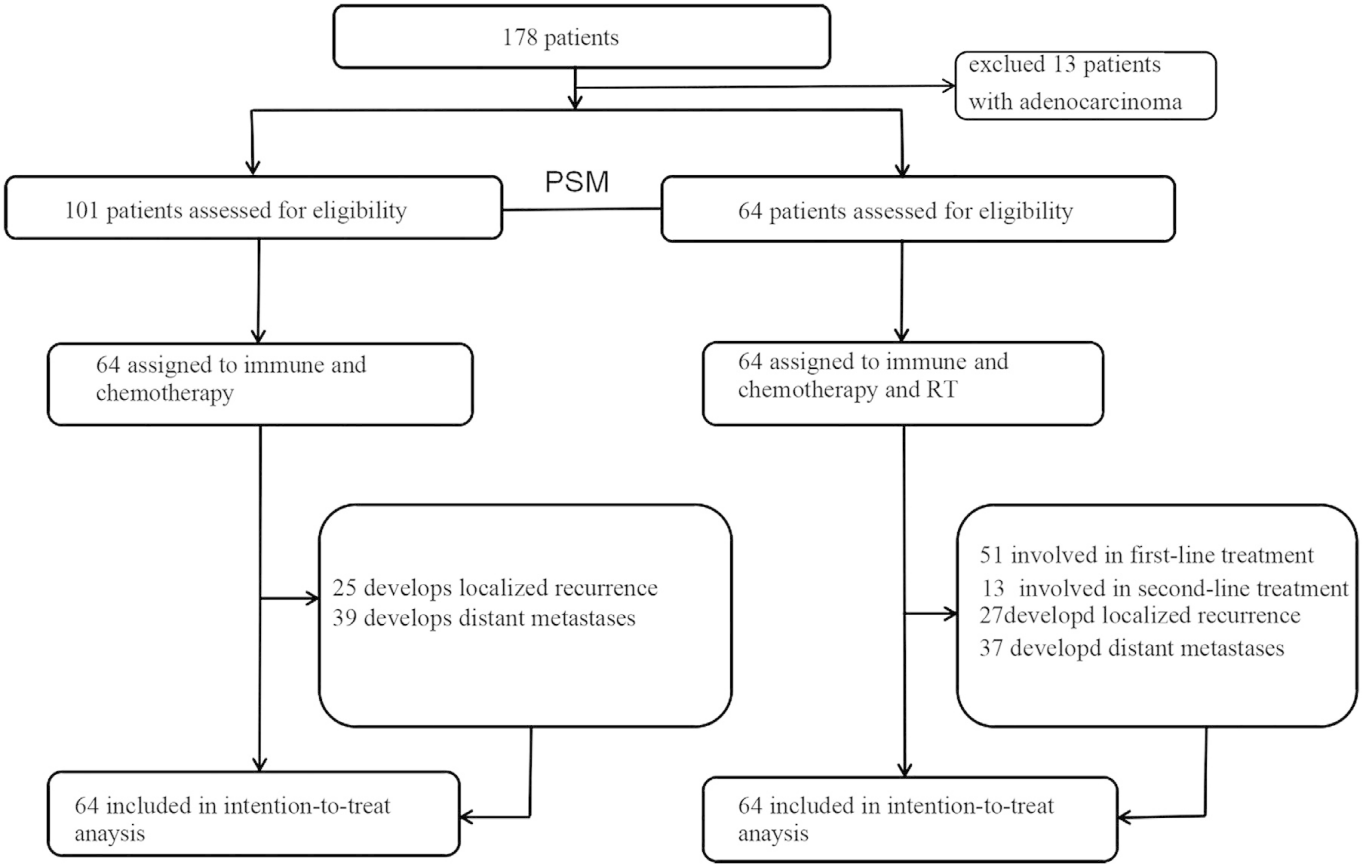
Research process. *Abbreviations*: PSM = propensity score matched; RT = radiation therapy.

This retrospective study analyzed clinical data from patients with metastatic or recurrent esophageal cancer who received first-line chemotherapy combined with immunotherapy at the First Affiliated Hospital of USTC Cancer Center between December 2019 and October 2023. Through electronic medical record screening, 178 cases meeting preliminary eligibility criteria were initially identified. After rigorous reevaluation against inclusion criteria, propensity score matching (PSM) was ultimately applied, yielding 128 well-matched cases for final analysis (Fig. 1). The ICIs + RT group received immunotherapy, chemotherapy, and RT, whereas the ICIs group received only immunotherapy and chemotherapy. The main inclusion criteria were as follows: (1) histologically confirmed diagnosis of ESCC; (2) clearly diagnosed metastatic or recurrent disease; (3) first-line treatment with chemotherapy combined with ICIs; and (4) an Eastern Cooperative Oncology Group performance status (ECOG) score of 0 or 1. The exclusion criteria encompassed active autoimmune diseases or other active previous malignancies requiring treatment.

Pretreatment examinations included hematologic and biochemical tests, esophagogastric biopsies, contrast-enhanced chest computed tomography (CT), neck/abdominal CT or ultrasound, positron emission tomography/CT (PET/CT), esophageal magnetic resonance imaging, and barium swallow studies. Tumor staging was performed in accordance with the Eighth edition of the American Joint Committee on Cancer Tumor-Node-Metastasis (TNM) staging system.

The study employed standardized oncological definitions for disease progression patterns: locoregional recurrence (LRR) encompassed tumor reappearance either at the primary site or within regional lymphatic drainage basins, while disease progression involving extraregional lymph node stations or systemic organs was classified as distant metastasis (DM). All patients in the ICIs group had no history of RT or endoscopic therapy. Seven patients in the ICIs + RT group had previously received neoadjuvant chemoradiotherapy or definitive chemoradiotherapy. Furthermore, all patients included in the study underwent systemic therapy combining paclitaxel- and platinum-based chemotherapy combined with ICIs (Table 1).

**Table 1:** Patients’ characteristics and treatment information before and after PSM*^,^†

		Before PSM				After PSM			
Characteristics	Level	Total (n = 165,%)	ICIs (n = 101,%)	ICIs+RT (n = 64,%)	*P* value	Total (n = 128,%)	ICIs (n = 64,%)	ICIs+RT (n = 64,%)	*P* value
Gender	Female	142 (86.06)	88 (87.13)	54 (84.38)	.619	18 (15.16)	8 (12.50)	10 (15.62	.611
	Male	23 (13.94)	13 (12.87)	10 (15.62)		110 (85.94)	56 (87.50)	54 (84.38)	
Age	<70	83 (50.30)	45 (44.55)	38 (59.38)	.064	67 (52.34)	29 (45.31)	38 (59.38)	.111
	≥70	82 (49.70)	56 (55.45)	26 (40.62)		61 (47.66)	35 (54.69)	26 (40.62)	
Location	Lower	15 (9.09)	7 (6.93)	8 (12.50)	.347	52 (40.62)	29 (45.31)	23 (35.94)	.225
	Middle	82 (49.70)	49 (48.51)	33 (51.56)		65 (50.78)	32 (50.00)	33 (51.56)	
	Upper	68 (41.21)	45 (44.55)	23 (35.94)		11 (8.59)	3 (4.69)	8 (12.50)	
Stage	Ⅲ-ⅣA	72 (43.64)	44 (43.56)	28 (43.75)	.981	53 (41.41)	25 (39.06)	28 (43.75)	.474
	ⅣB	93 (56.36)	57 (56.44)	36 (56.25)		75 (58.59)	39 (60.94)	36 (56.25)	
ECOG	0	79 (47.88)	40 (39.60)	39 (60.94)	.008	74 (57.81)	35 (54.69)	39 (60.94)	.719
	1	86 (52.12)	61 (60.40)	25 (39.06)		54 (42.19)	29 (45.31)	25 (39.06)	

*Abbreviations:* ECOG = Eastern Cooperative Oncology Group; ICIs = immune checkpoint inhibitors; PSM = propensity score matched; RT = radiation therapy.

⁎ All patients received paclitaxel-based therapy combined with platinum-based agents.

† Patients in the ICIs+RT group received a median radiation dose of 50 Gy.

The study adhered to the Declaration of Helsinki and received approval from the institutional review board, and because of its retrospective nature, informed consent was waived.

### Treatment and follow-up

The most commonly employed chemotherapy regimens consisted of systemic therapy combining paclitaxel with platinum-based compounds. The primary immunotherapeutic agents were anti-PD-1 antibodies, specifically including camrelizumab, tislelizumab, and pembrolizumab, which were administered either concurrently with or sequentially to chemotherapy. Radiation therapy was performed using intensity modulated RT or volumetric-modulated arc therapy with an energy of 6 MV. The RT followed the involved-field irradiation principle. The gross tumor volume of the primary tumor (GTVp) was defined as the primary esophageal tumor, and the gross tumor volume of lymph nodes was defined as positive lymph nodes confirmed by contrast-enhanced CT, PET/CT scans, or cytology. For the locoregional field, GTVp of the primary lesion was expanded with 3 cm margins proximally and distally, GTVp and gross tumor volume of lymph nodes along with a 0.8 cm radial margin, to generate the clinical target volume. The planning gross tumor volume (PGTV) and the planning target volume (PTV) exceeded the GTV or clinical target volume by 5 mm in 3 dimensions. For patients with distant metastases, positive cervical and abdominal lymph nodes were typically included in the radiation field, while local treatment was optional for distant organs such as the lungs, liver, and bones. For locoregional lesions field (36 cases, 56.3%), the prescribed doses for PGTV or PTV were 40 to 60 Gy in 20 to 30 fractions using simultaneously integrated boost technique on 5 consecutive days per week (Table E1). Among 28 cases (43.8%) with DM lesions, the 6 bone and soft-tissue metastasis cases (9.3%) received 30 to 60 Gy in 10 to 28 fractions, reflecting the customized approach characteristic of palliative treatment. The substantial variation in radiation doses (20-50 Gy) and fractionation schedules (4-20 fractions) for the 7 lung metastasis cases (10.9%) likely indicates the utilization of diverse techniques including stereotactic body RT. The remaining cases comprised 8 (12.5%) with metastases to other sites treated with 24 to 48 Gy in 3 to 15 fractions, and 7 (10.9%) with nonregional lymph node involvement receiving 15 to 45 Gy in 5 to 15 fractions (Table E1).

Radiation therapy mainly targeted the primary tumor, mediastinal metastatic lymph nodes, nonregional lymph nodes, and distant metastatic organs, using intensity modulated RT or volumetric-modulated arc therapy with an energy of 6 MV. The patients were followed up every 3 months for the first 2 years, every 6 months for the next 2 years, and annually after 5 years. Clinical examinations included routine blood tests and tumor markers, contrast-enhanced chest CT, neck/abdominal CT or ultrasound, barium swallow studies, and PET/CT if indicated.

### Endpoints and statistical analysis

Intergroup covariate comparisons were performed using Pearson’s χ² test or Fisher’s exact probability test for nominal variables. To address potential confounding bias, PSM analysis was implemented. Propensity scores were derived through multivariable logistic regression incorporating clinically relevant covariates: gender, chronological age, ECOG performance status, and tumor staging parameters. Applying a nearest-neighbor matching algorithm with a caliper width of 0.02 SDs and 1:1 matching ratio, 2 well-balanced cohorts were successfully generated, each comprising 64 matched cases. The primary endpoint of this study was the OS of the included patients. Secondary endpoints included the PFS, safety, and tolerability. Continuous variables were summarized using descriptive statistics, including mean, SD, median, and range. Categorical variables were tabulated as frequencies and percentages. The OS was calculated from the date of recurrent/metastatic diagnosis until death or censoring, while the PFS was measured from the date of recurrent/metastatic diagnosis to disease progression, death, or censoring. The Kaplan-Meier method was used to estimate PFS and OS. Univariate and multivariate Cox proportional-hazards regression models were applied to identify significant prognostic factors affecting PFS and OS. Variables with *P* ≤ .05 in univariate analysis were included in the multivariate analysis. Differences in PFS and OS between groups were evaluated using the log-rank test. Statistical analyses were performed using R (version 4.1.3).

## Results

### Survival

**Figure 2:**
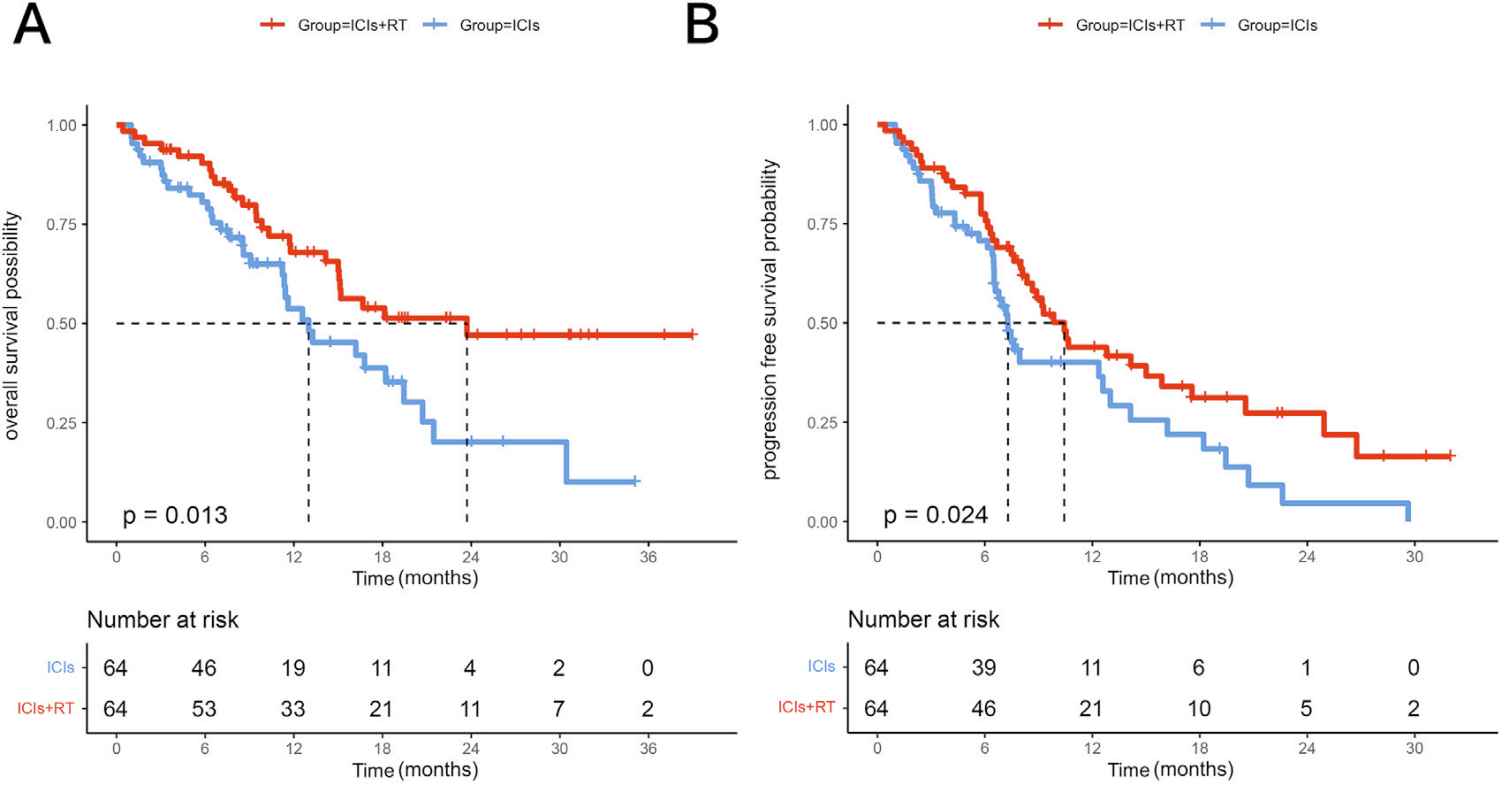
Overall survival (OS) and progression-free survival (PFS) of ICIs + RT group and ICIs group after PSM. (A) OS of ICIs + RT group and ICIs group. (B) PFS of ICIs+RT group and ICIs group. *Abbreviations:* ICIs = immune checkpoint inhibitors; RT = radiation therapy.

A summary of the demographic characteristics and therapeutic information before and after PSM is presented in Table 1. The majority of the patients were male (85.9%). In the ICIs + RT group, 60.9% of the patients had an ECOG performance status of 0, compared with 54.7% in the ICIs group (*P* = .791). A substantial proportion of the patients (58.6%) exhibited advanced-stage IVB disease. The accompanying table presents a comprehensive overview of the study population’s baseline characteristics and treatment details, emphasizing the balanced representation of the 2 groups following PSM. A total of 60 deaths were observed in the course of the analysis. The ICIs + RT group exhibited a mortality rate of 40.6% (n = 26), while the ICIs group demonstrated a mortality rate of 53.1% (n = 34). The median OS (mOS) was 23.71 months in the ICIs + RT group and 13.00 months in the ICIs group (hazard ratio [HR], 0.53; 95% CI, 0.31-0.88; *P* = .013) (Fig. 2A). The median PFS (mPFS) was 10.43 months in the ICIs + RT group and 7.27 months in the ICIs group (HR, 0.61; 95% CI, 0.39-0.94; *P* = .024) (Fig. 2B).

To investigate the impact of the RT timing on patient prognosis, the ICIs + RT group was divided into 2 subgroups, namely subgroup A, comprising patients who received RT before progression, and subgroup B, comprising patients who received RT after progression. The mOS was 18.14 months for subgroup A and not reached for subgroup B (HR, 0.62; 95% CI, 0.23-1.6; *P* = .326) (Fig. E1).

**Figure 3:**
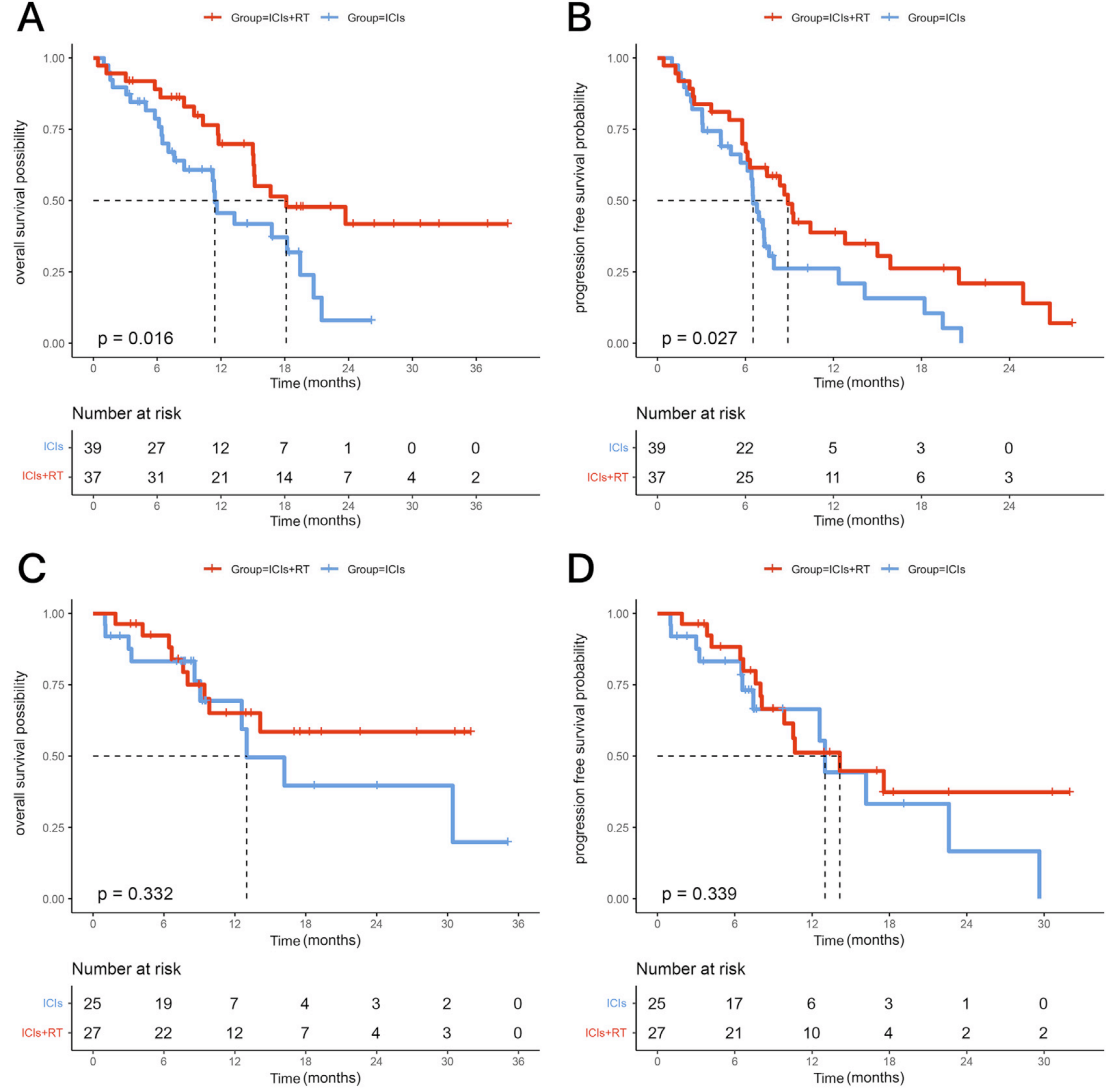
Overall survival (OS) and progression-free survival (PFS) for patients with distant metastasis and locoregional recurrence in ICIs + RT and ICIs groups. (A) OS of ICIs + RT group and ICIs group in distant metastasis patients. (B) PFS of ICIs + RT group and ICIs group in distant metastasis patients. (C) OS of ICIs + RT group and ICIs group in locoregional recurrences patients. (D) PFS of ICIs + RT group and ICIs group in locoregional recurrences patients. *Abbreviations:* ICIs = immune checkpoint inhibitors; RT = radiation therapy.

Further analysis was conducted to determine the value of adding RT to the treatment regimen for 2 distinct patient subgroups, namely those with only local recurrence and those with DM. A total of 42.2% of the patients in the ICIs + RT group and 39.1% of the patients in the ICIs group experienced only local recurrence. For the patients with DM, the mOS was 18.14 months in the ICIs + RT group and 11.42 months in the ICIs group (HR, 0.74, 95% CI, 0.43-1.28, *P* = .016) (Fig. 3A). The mPFS was 8.89 months in the ICIs + RT group and 6.52 months in the ICIs group (HR, 0.48; 95% CI, 0.30-0.77, *P* = .027) (Fig. 3B). For the patients with LRR, the mOS in the ICIs + RT group was not reached, while the ICIs group had a median OS of 13.00 months (HR, 0.64; 95% CI, 0.26-1.58, *P* = .332) (Fig. 3C). The mPFS was 14.15 months in the ICIs + RT group and 13.00 months in the ICIs group, but without reaching statistical significance (HR, 0.68; 95% CI, 0.31-1.50, *P* = .339) (Fig. 3D).

**Figure 4:**
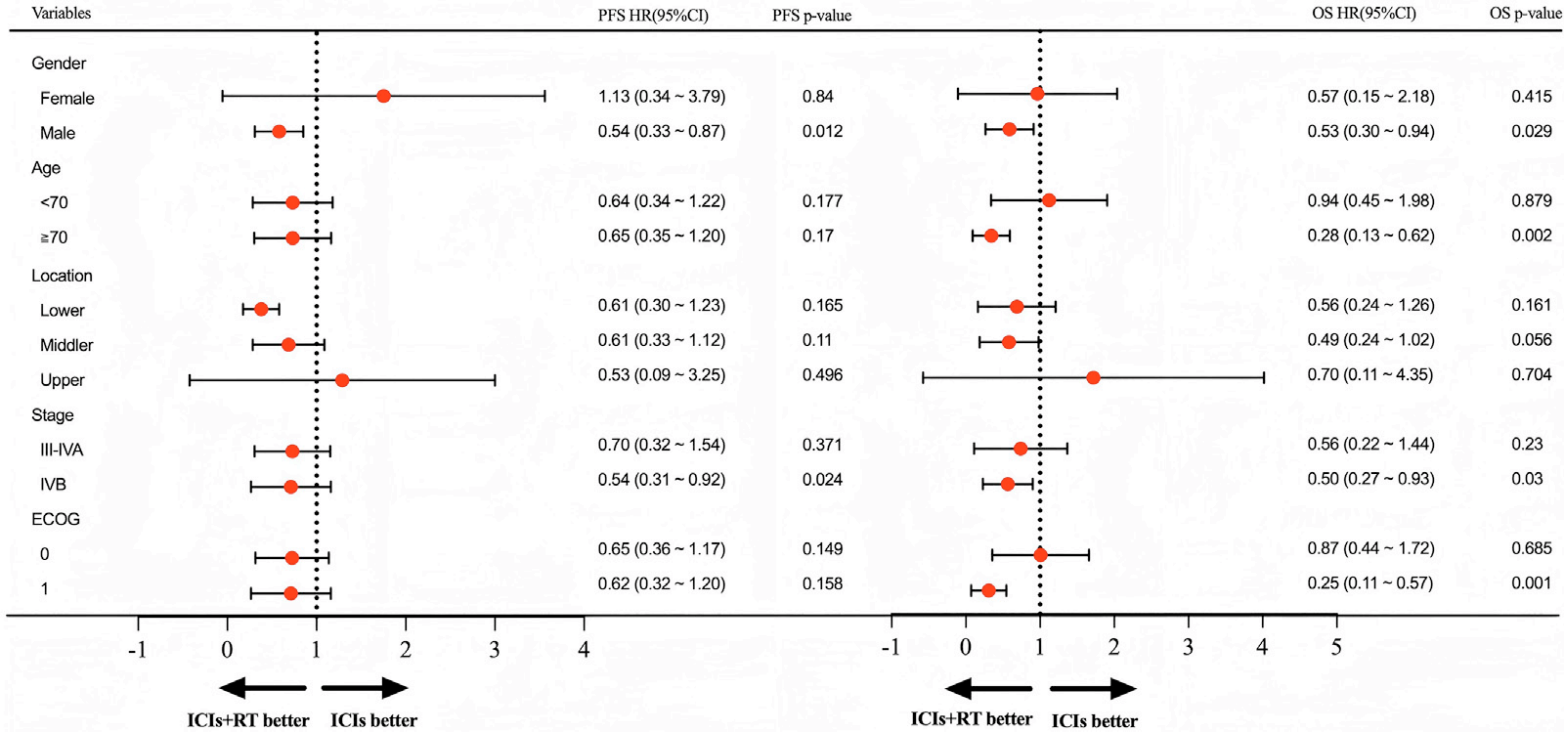
Hazard ratios (HRs) for subgroups of overall survival (OS) and progression-free survival (PFS) comparing ICIs + RT group to ICIs group. *Abbreviations:* ECOG = Eastern Cooperative Oncology Group; ICIs = immune checkpoint inhibitors; RT = radiation therapy.

Subgroup analyses were conducted for both OS and PFS to explore the potential benefits of combining RT with immunochemotherapy (Fig. 4). As demonstrated in the forest plot, male patients (*P* = .029), those with stage IVB disease (*P* = .03) exhibited improved OS with the addition of RT to immunochemotherapy. For PFS, male patients (*P* = .012) and those with stage ⅣB disease (*P* = .024) benefited from the combination treatment.

The univariate analysis indicated that the treatment modality was a potential predictor for both OS and PFS (Table 2). The HR for the OS was 0.53 (95% CI, 0.31-0.88; *P* = .015), while the HR for the PFS was 0.61 (95% CI, 0.39-0.94; *P* = .026). Furthermore, disease stage (HR, 2.07; 95% CI, 1.28-3.34; *P* = .003) and radiation field (HR, 0.48; 95% CI, 0.30-0.77; *P* = .003) were identified as predictors for PFS. After adjusting for multiple covariates, treatment modality (HR, 0.60; 95% CI, 0.38-0.93; *P* = .022) remained significantly associated with PFS (Table E2). These results indicate that the combination of RT with ICIs is particularly beneficial for male patients and those with advanced stage or DM, highlighting the importance of treatment modality and disease stage in predicting patient outcomes.

**Table 2:** Univariate analysis for factors associated with PFS and OS

Variables	Univariable analysis							Univariable analysis				
	PFS	β	SE	Z	*P*	HR (95% CI)	OS	β	SE	Z	*P*	HR (95% CI)
Treatment												
ICIs						1.00 (Reference)						1.00 (Reference)
ICIs+RT		-0.50	0.22	-2.23	0.026	0.61 (0.39-0.94)		-0.64	0.26	-2.44	0.015	0.53 (0.31-0.88)
Gender												
Female						1.00 (Reference)						1.00 (Reference)
Male		0.26	0.30	0.85	0.397	1.29 (0.71-2.35)		0.01	0.36	0.03	0.978	1.01 (0.50-2.05)
Age												
≥70						1.00 (Reference)						1.00 (Reference)
<70		0.28	0.22	1.28	0.200	1.33 (0.86-2.05)		0.09	0.26	0.37	0.715	1.10 (0.66-1.83)
Location												
Lower						1.00 (Reference)						1.00 (Reference)
Middle		0.08	0.23	0.34	0.730	1.08 (0.69-1.71)		0.03	0.27	0.12	0.908	1.03 (0.60-1.76)
Upper		-0.07	0.45	-0.15	0.877	0.93 (0.39-2.24)		0.08	0.49	0.15	0.878	1.08 (0.41-2.84)
Stage												
Ⅲ-ⅣA						1.00 (Reference)						1.00 (Reference)
ⅣB		0.73	0.24	2.99	0.003	2.07 (1.28-3.34)		0.40	0.28	1.41	0.157	1.49 (0.86-2.60)
ECOG												
0						1.00 (Reference)						1.00 (Reference)
1		0.22	0.22	0.99	0.321	1.25 (0.81-1.92)		0.16	0.26	0.60	0.547	1.17 (0.70-1.94)
Site												
Distant						1.00 (Reference)						1.00 (Reference)
Local		-0.73	0.24	-3.01	0.003	0.48 (0.30-0.77)		-0.30	0.28	-1.08	0.280	0.74 (0.43-1.28)

*Abbreviations:* ECOG = Eastern Cooperative Oncology Group; HR = hazard ratio; ICIs = immune checkpoint inhibitors; OS = overall survival; PFS = progression-free survival; PSM = propensity score matched; RT = radiation therapy.

### Safety

The most common treatment-related adverse events were leukopenia, treatment-related esophagitis, and neutropenia (Table 3). The ICIs + RT group experienced higher incidence rates of these adverse events compared with the ICIs group. This table highlights the increased incidence of leukopenia and treatment-related esophagitis in the ICIs + RT group compared with the ICIs group. Grade 3-4 treatment-related adverse events occurred in 20 of 64 (31%) patients in the ICIs + RT group and 16 of 64 (25%) patients in the ICIs group. The incidence of neutropenia was also higher in the ICIs + RT group. In addition, there was 1 case of treatment-related esophageal fistula in the ICIs + RT group. No treatment-related deaths were reported by the date of publication.

**Table 3:** Summary of treatment-related adverse events

	ICIs group (n = 64) (%)				ICIs and RT group (n = 64) (%)			
	Grade 1-2	Grade 3	Grade 4	Grade 5	Grade 1-2	Grade 3	Grade 4	Grade 5
Leukopenia	12 (19)	7 (11)	0	0	14 (22)	8 (13)	1 (2)	0
Neutropenia	6 (9)	7 (11)	0	0	7 (11)	5 (8)	1 (2)	0
Esophagitis	1 (2)	0	0	0	17 (27)	2 (3)	1 (2)	0
Pneumonia	3 (5)	1 (2)	0	0	6 (9)	2 (3)	0	0
Thrombocytopenia	2 (3)	1 (2)	0	0	3 (5)	0	0	0
Hypothyroidism	1 (2)	0	0	0	2 (3)	0	0	0

*Abbreviations:* ICIs = immune checkpoint inhibitors; RT = radiation therapy.

## Discussion

In clinical practice, the preferred treatment strategy for recurrent or metastatic esophageal cancer is systemic therapy.^13^^,^^14^ Our retrospective study indicated that adding RT to chemoimmunotherapy could significantly improve the survival of patients with recurrent/metastatic esophageal cancer. In addition, the combination of ICIs and RT was found to be safe and tolerable.

The KEYNOTE-590 study found that patients with advanced esophageal cancer receiving first-line chemoimmunotherapy achieved the mPFS and mOS of 6.3 and 12.6 months, respectively. Similarly, the the Evaluation of Safety and Clinical Outcome of Recombinant T-cell Therapy-1 (ESCORT-1) study reported the mPFS and mOS of 6.9 and 15.3 months, respectively.^15^^,^^16^ In the present study, the patients who underwent RT in combination with immunochemotherapy exhibited the mPFS and mOS of 10.43 (95% CI, 8.54-12.32) and 23.71 months (95% CI, NA), respectively. The results suggest that RT may confer a survival benefit when combined with immunochemotherapy.

A number of studies have reported disparate OS outcomes for patients with locoregionally recurrent esophageal cancer treated with RT or chemoradiotherapy, with reported values ranging from 7.0 to 24.3 months.^16^, ^17^, ^18^, ^19^, ^20^, ^21^, ^22^ However, the value of this treatment strategy in metastatic disease is unclear. The present study demonstrated that, in comparison with systemic therapy alone, the combination of ICIs and RT markedly prolonged both PFS and OS in patients presenting with DM. The OS and PFS for the ICIs + RT group were 18.14 (95% CI, 18.29-28.49) and 8.89 months (95% CI, 8.95-15.15), respectively. However, in the subgroup with only local recurrence, adding RT to chemoimmunotherapy did not confer a significant survival benefit. This finding is at odds with previous studies and challenges our fundamental understanding. One plausible explanation could be the insufficient sample size. In LRR cohort, although there was no statistically significant difference between the 2 groups, the ICIs + RT group demonstrated a trend favoring improved outcomes. Enlarging the sample size and extending the follow-up period may yield statistically significant results. An alternative explanation could be the radiation-induced damage to immune cells.^23^ Further analysis showed that out of 27 only local recurrence/progression patients, 21 (77%) received more radical RT, typically given concurrently with chemotherapy and immunotherapy. This form of RT was predominantly aimed at definitive doses (50-60 Gy/25-30 fractions). For patients with distant metastases, RT was most frequently administered in conjunction with ICIs maintenance therapy, following chemoimmunotherapy. The majority of patients with distant metastases received a short course of RT, typically comprising ≤10 fractions, and were more likely to receive hypofractionated RT. The toxic side effects of triple therapy and the sustained damage to lymphocytes from long-course RT might impair the benefits of ICIs, which could reasonably explain the discrepancy between our findings and those from the chemotherapy era. In addition, the ESO-Shanghai 13 study focused on patients with oligometastatic esophageal cancer who received systemic therapy combined with local therapy. The mPFS for the systemic and local therapy groups was 15.3 (95% CI, 10.1-20.5) and 6.4 months (95% CI, 5.2-7.6), respectively, indicating that patients with oligometastatic disease benefit more from aggressive local therapy.^24^ These studies indicate that, in comparison with chemoradiotherapy, the combination of immunotherapy and RT requires a more meticulous evaluation of RT dose fractionation schemes and the timing of administration to achieve optimal therapeutic outcomes.

The optimal radiation dose for recurrent metastatic esophageal cancer remains controversial. Guttmann et al^25^ conducted an observational cohort study of esophageal cancer patients and found that chemotherapy combined with a definitive radiation dose (5040 cGy) extended OS compared with chemotherapy alone, while chemotherapy combined with palliative radiation doses (<5040 cGy) did not significantly improve OS. Similarly, Li et al^26^ demonstrated that a biologically effective dose 10 of ≥60 Gy significantly extended median OS compared with biologically effective dose 10 <60 Gy (16 vs 10 months, *P* = .033). In our study, we conducted further analysis to determine the impact of different radiation doses on primary esophageal lesions and metastatic mediastinal lymph nodes. The ICIs + RT group was divided into 2 subgroups based on the radiation doses administered, with 1 subgroup receiving ≥50 Gy and the other subgroup receiving <50 Gy. The results showed no statistically significant differences in OS and PFS between the 2 subgroups (Figs. E2A, B). This outcome may be because of the potential synergistic immunologic effects and increased toxicity accompanying the combination of RT and ICIs in esophageal cancer. Wu et al^27^ reported that patients who received RT within 90 days before or after ICIs treatment had higher survival rates than those who received RT outside this 90-day window, suggesting that RT should be part of first-line treatment rather than a palliative measure after disease progression. The disparate patient populations between studies may be a contributing factor to the observed discrepancies in results. However, in our study, regardless of whether patients received first- or second-line treatment, the core of systemic therapy was immunotherapy. The antitumor immune activation effects of RT suggest that low-dose RT combined with ICIs could effectively control tumor metastasis by mobilizing innate and adaptive immunity, downregulating immunosuppressive factors such as Transforming Growth Factor-βeta (TGF-β), and partially reversing and delaying immune resistance.^28^

Another concern with combination therapy is toxicity. In a phase 1b trial of RT (60 Gy in 30 fractions) combined with camrelizumab as first-line treatment for locally advanced ESCC, 10 out of 19 patients (53%) experienced grade 3-4 adverse events.^29^ In the EC-CRT-001 trial, a phase 2 study of toripalimab combined with definitive chemoradiotherapy (50.4 Gy in 28 fractions) for locally advanced ESCC, 36 out of 42 patients (86%) experienced grade 3-4 adverse events, including 17 patients (41%) with nonhematological adverse events, and 1 patient (2%) died from treatment-related pneumonia.^30^ A relatively high incidence of toxicity has been observed with triple therapy. In our study of recurrent or metastatic esophageal cancer, the ICIs + RT group exhibited a low incidence of grade 3 or higher treatment-related adverse events (31%) (Table 3) and no treatment-related deaths. The limitations of this study, including its retrospective design and the small number of patients, could have resulted in an underestimation of the actual toxicity levels. Therefore, further studies with larger samples and a prospective design are needed to fully assess the true adverse effects of this combination therapy.

Our study has several limitations that should be addressed. First, the results are limited by the retrospective design and the small sample size. Although the only significantly different variable between the ICIs + RT group and the ICIs group was the line of chemoradiotherapy, inherent selection bias remains. Second, the lack of essential biomarker information, such as PD-L1 expression, is a significant limitation. Finally, a notable limitation of this study is its exclusive focus on patients with squamous cell carcinoma, which may limit the generalizability of the findings to adenocarcinoma or other non- squamous cell carcinoma subtypes.

In conclusion, our study indicates that adding RT to chemoradiotherapy significantly improves the OS of patients with locally advanced recurrent and metastatic esophageal cancer with good safety. We acknowledge that our results require validation and further improvements in larger, prospective, multicenter studies.

## Disclosures

The authors declare that they have no known competing financial interests or personal relationships that could have appeared to influence the work reported in this paper.

## Appendix Supplementary materials

Supplementary material 20250504 Image, application 1

## Footnotes

Supplementary material associated with this article can be found in the online version at doi:10.1016/j.adro.2025.101890.
