# Supplementary material for: Survival Benefit of Combined Chemoimmunotherapy and Radiation Therapy in Patients with Recurrent or Metastatic Esophageal Cancer
Source: Adv Radiat Oncol. 2025 Aug 22;10(11):101890. doi: 10.1016/j.adro.2025.101890 (PMC12495256; doi:10.1016/j.adro.2025.101890)
Supplement: Supplementary material 20250504 [file mmc1.docx]

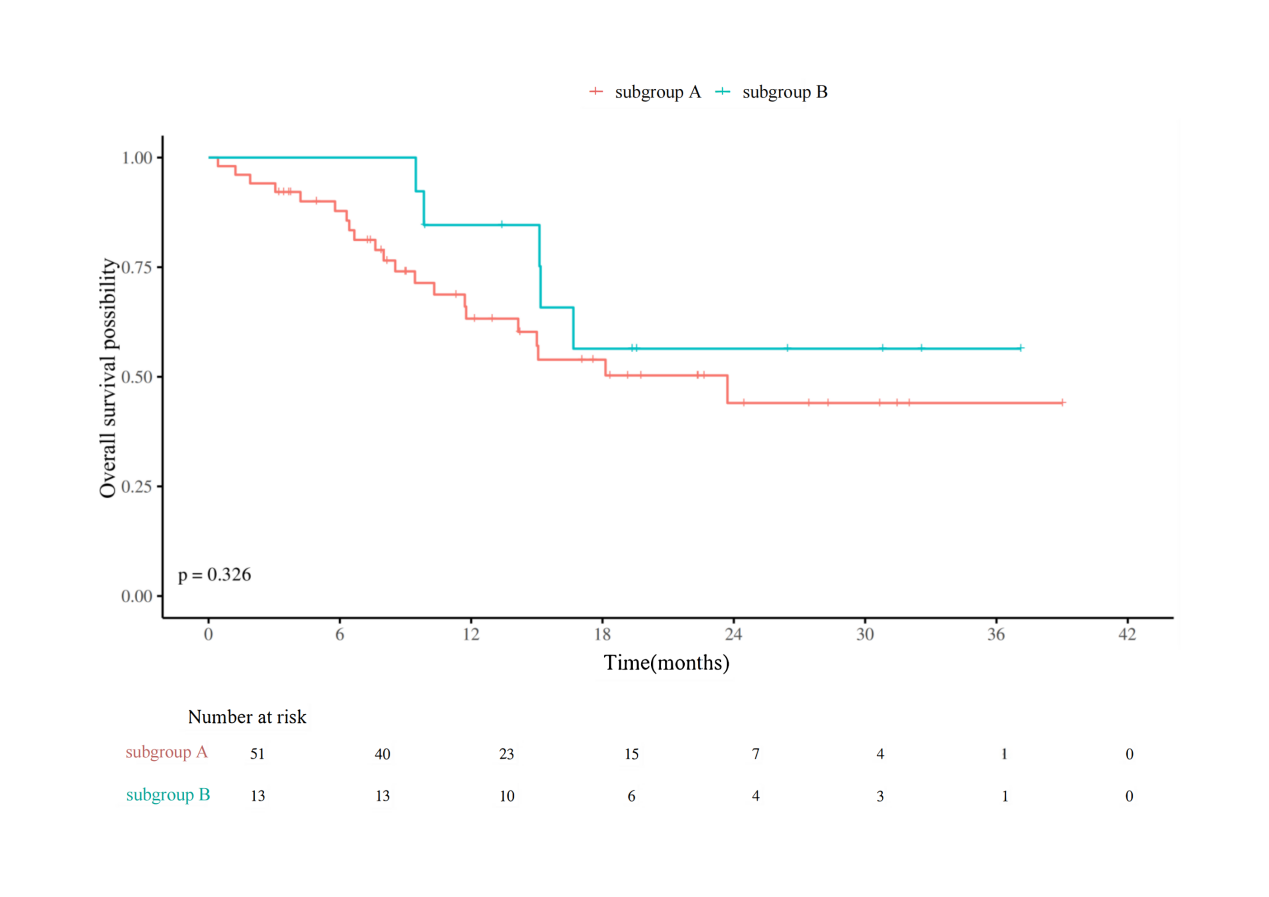


**Supplementary Fig.1：**OS of subgroup A (RT before progression) and subgroup B (RT after progression).


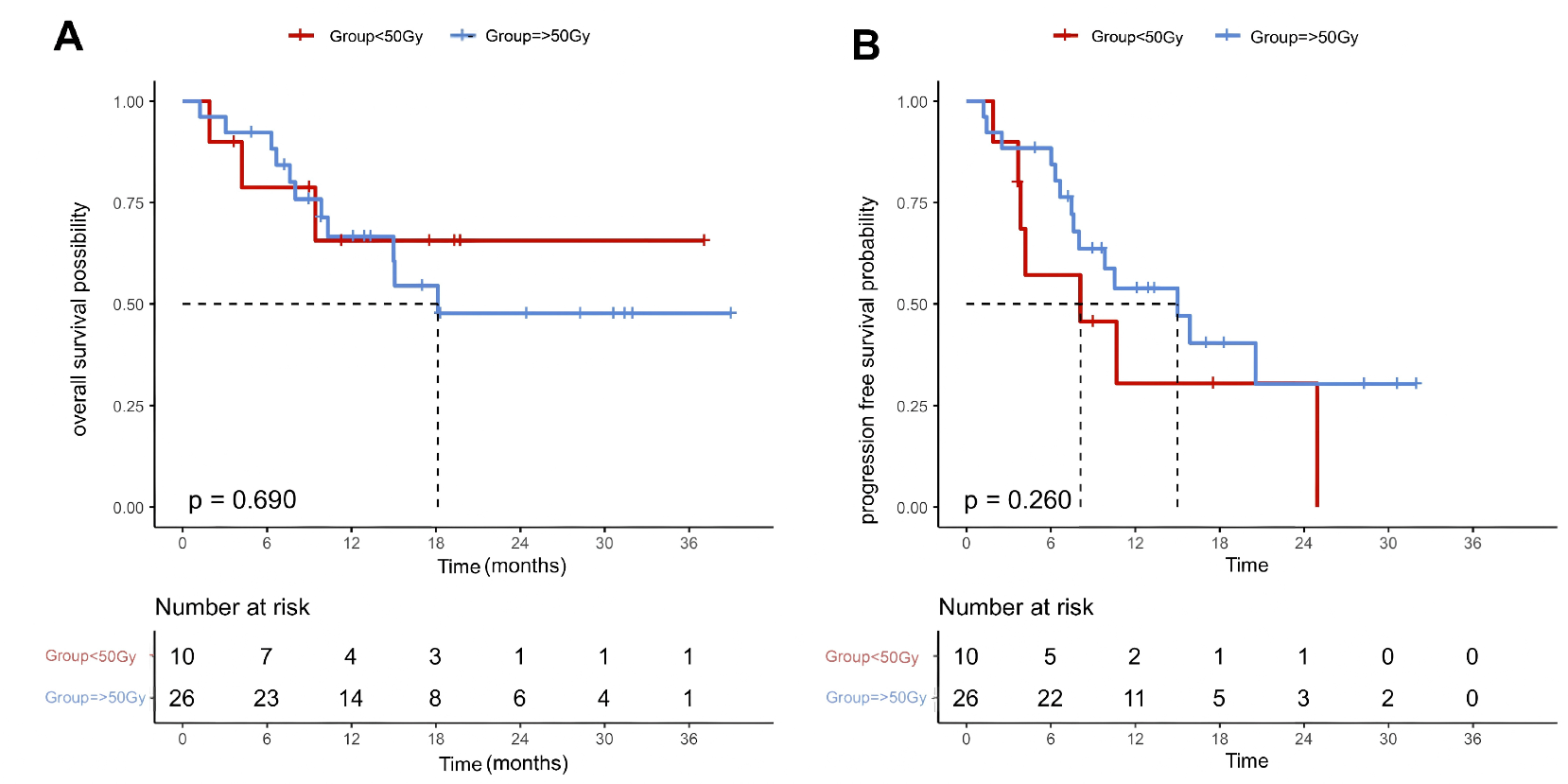


**Supplementary Fig.2:**Overall survival (A) and Progression-free survival (B) with radiotherapy dose to primary esophageal and medi-regional lymph nodes in the RT group.

**Supplementary Table 1.**Radiotherapy Dose Fractionation Regimens in the ICIs+RT group

| Radiation field | No(%) | | |
| --- | --- | --- | --- |
|  | ICIs+RT group  (n=64,%) | **Dose Range for Radiation Therapy**(Gy) | Number of fractions(f) |
| **Locoregional lesions** | 36(56.3) | 40-60 | 20-30 |
| Distant metastasis lesions | 28(43.8) |  |  |
| **Bone and soft tissue** | 6(9.3) | 30-60 | 10-28 |
| Lung | 7(10.9) | 20-50 | 4-20 |
| Others | 8(12.5) | 24-48 | 3-15 |
| Non-regional lymph nodes | 7(10.9) | 15-45 | 5-15 |

**Supplementary Table 2**. multivariable analysis for factors associated with PFS and OS

| Variables |  | Multivariable analysis | | | | |  | Multivariable analysis | | | | |
| --- | --- | --- | --- | --- | --- | --- | --- | --- | --- | --- | --- | --- |
|  | PFS | β | S.E | Z | *P* | HR (95%CI) | OS | β | S.E | Z | *P* | HR (95%CI) |
| Treatment |  |  |  |  |  |  |  |  |  |  |  |  |
| ICIs |  |  |  |  |  | 1.00 (Reference) |  |  |  |  |  |  |
| ICIs+RT |  | -0.52 | 0.22 | -2.30 | 0.022 | 0.60 (0.38 ~ 0.93) |  |  |  |  |  |  |
| Gender |  |  |  |  |  |  |  |  |  |  |  |  |
| Female |  |  |  |  |  |  |  |  |  |  |  |  |
| Male |  |  |  |  |  |  |  |  |  |  |  |  |
| Age |  |  |  |  |  |  |  |  |  |  |  |  |
| ≥70 |  |  |  |  |  |  |  |  |  |  |  |  |
| ＜70 |  |  |  |  |  |  |  |  |  |  |  |  |
| Location |  |  |  |  |  |  |  |  |  |  |  |  |
| Lower |  |  |  |  |  |  |  |  |  |  |  |  |
| Middle |  |  |  |  |  |  |  |  |  |  |  |  |
| Upper |  |  |  |  |  |  |  |  |  |  |  |  |
| ECOG |  |  |  |  |  |  |  |  |  |  |  |  |
| 0 |  |  |  |  |  |  |  |  |  |  |  |  |
| 1 |  |  |  |  |  |  |  |  |  |  |  |  |
